# Supplementary figures and images for: Recognition of discrete export signals in early flagellar subunits during bacterial type III secretion
Source: eLife. 2022 Mar 3;11:e66264. doi: 10.7554/eLife.66264 (PMC8983047; doi:10.7554/eLife.66264)

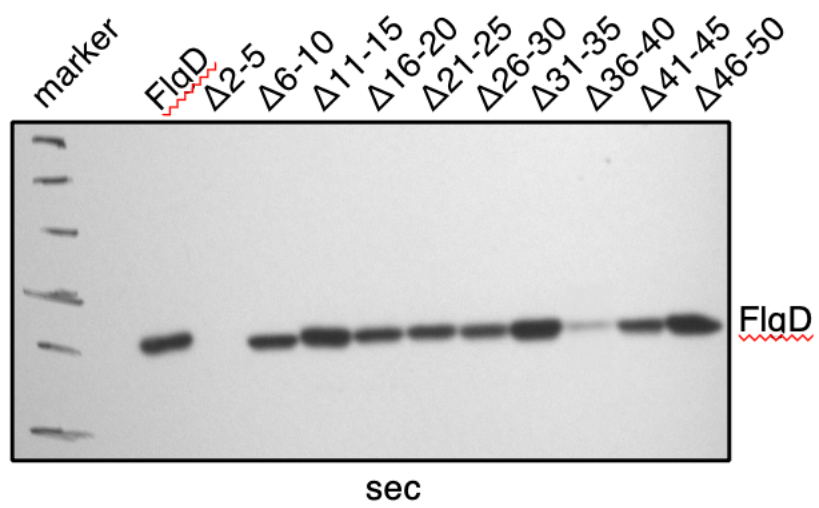

Supplement: Figure 1—source data 1. [file elife-66264-fig1-data1.pdf]

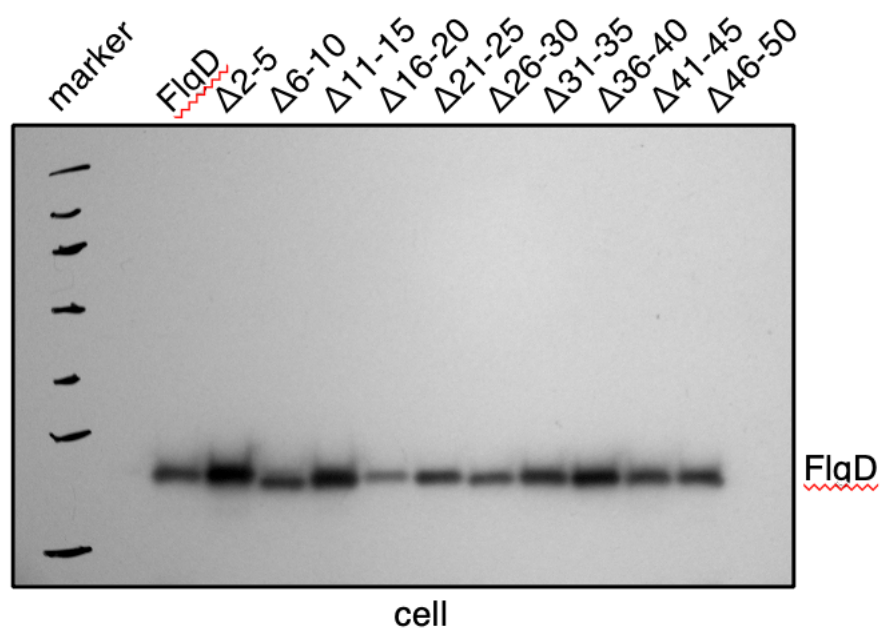

Supplement: Figure 1—source data 2. [file elife-66264-fig1-data2.pdf]

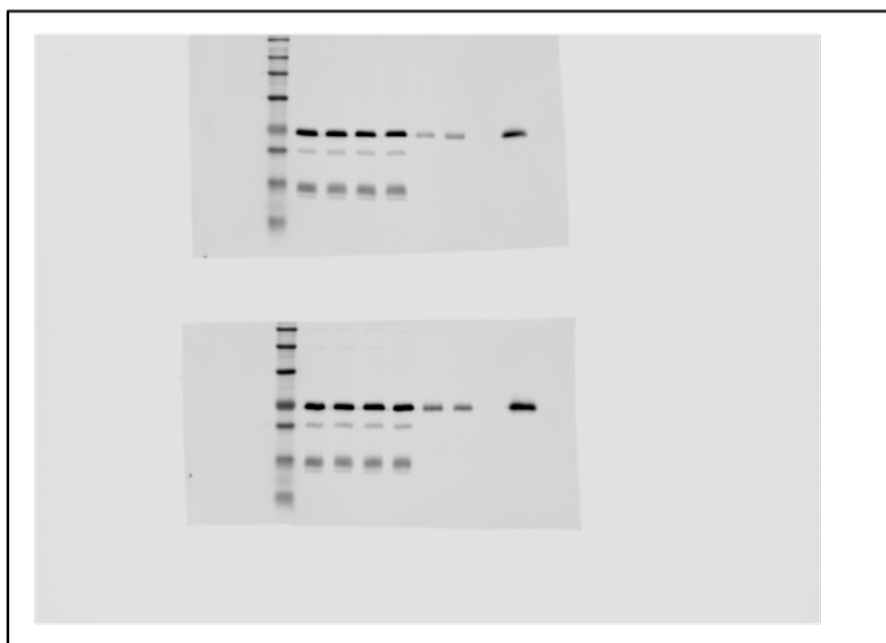

Supplement: Figure 1—source data 3. [file elife-66264-fig1-data3.pdf]

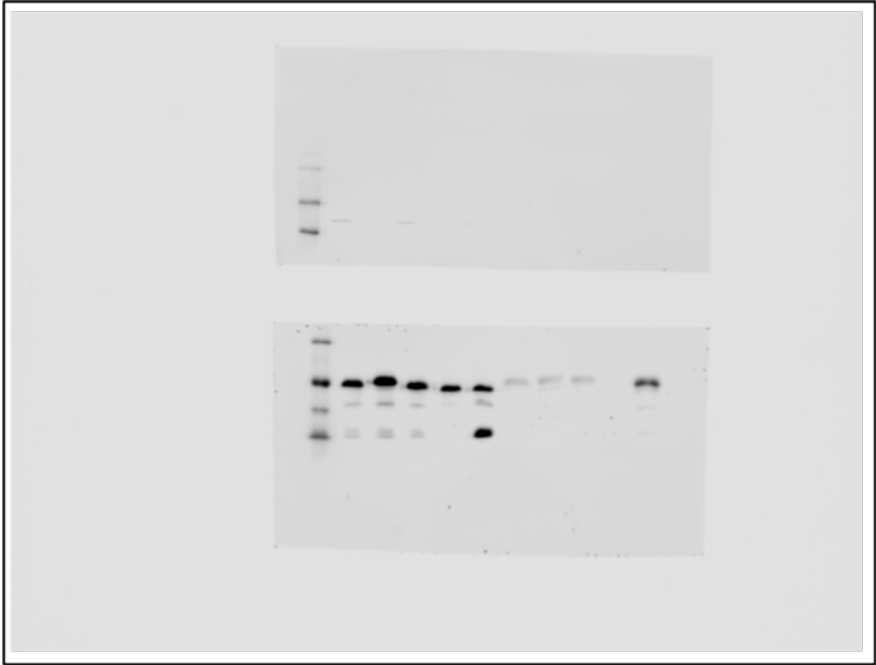

Supplement: Figure 1—source data 4. [file elife-66264-fig1-data4.pdf]

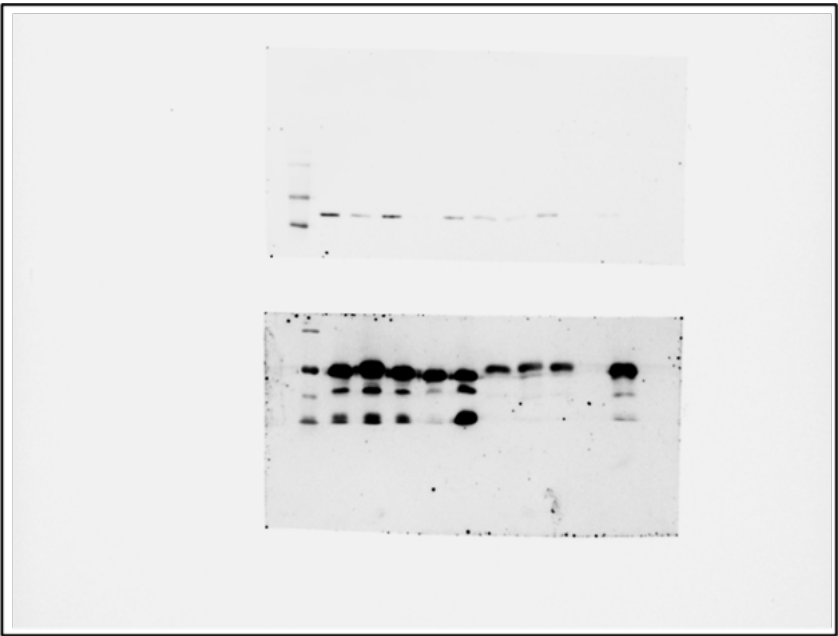

Supplement: Figure 1—source data 5. [file elife-66264-fig1-data5.pdf]

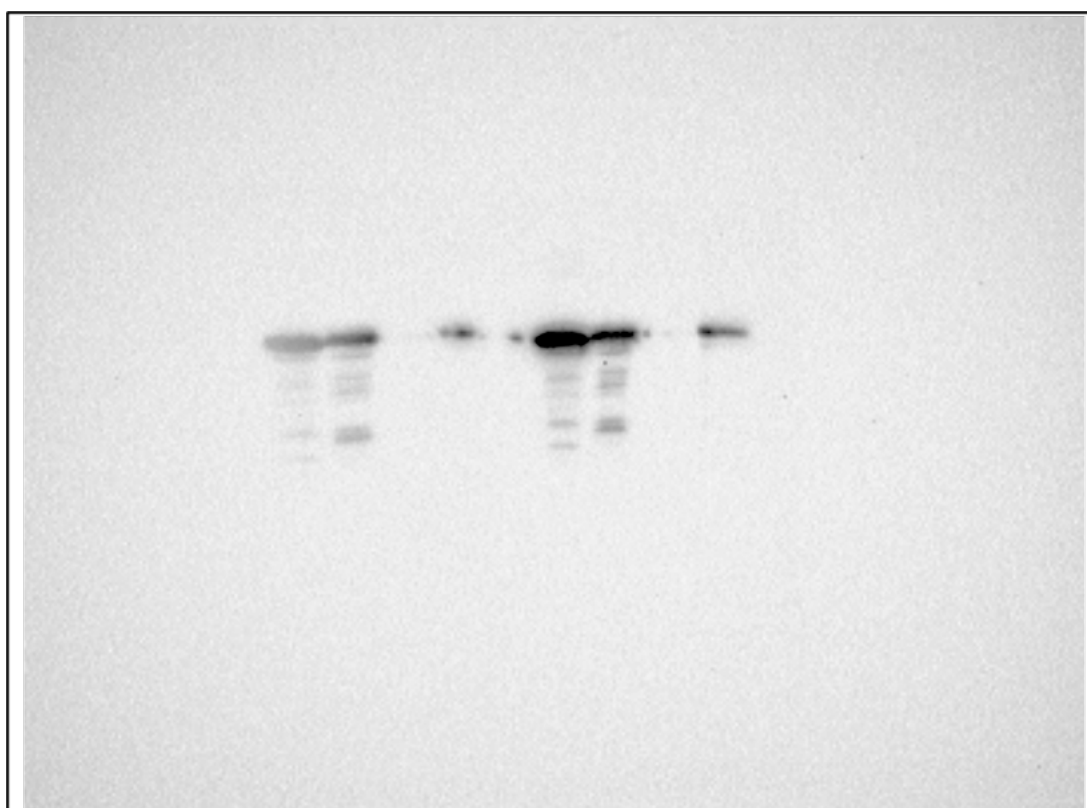

Supplement: Figure 1—figure supplement 3—source data 1. [file elife-66264-fig1-figsupp3-data1.pdf]

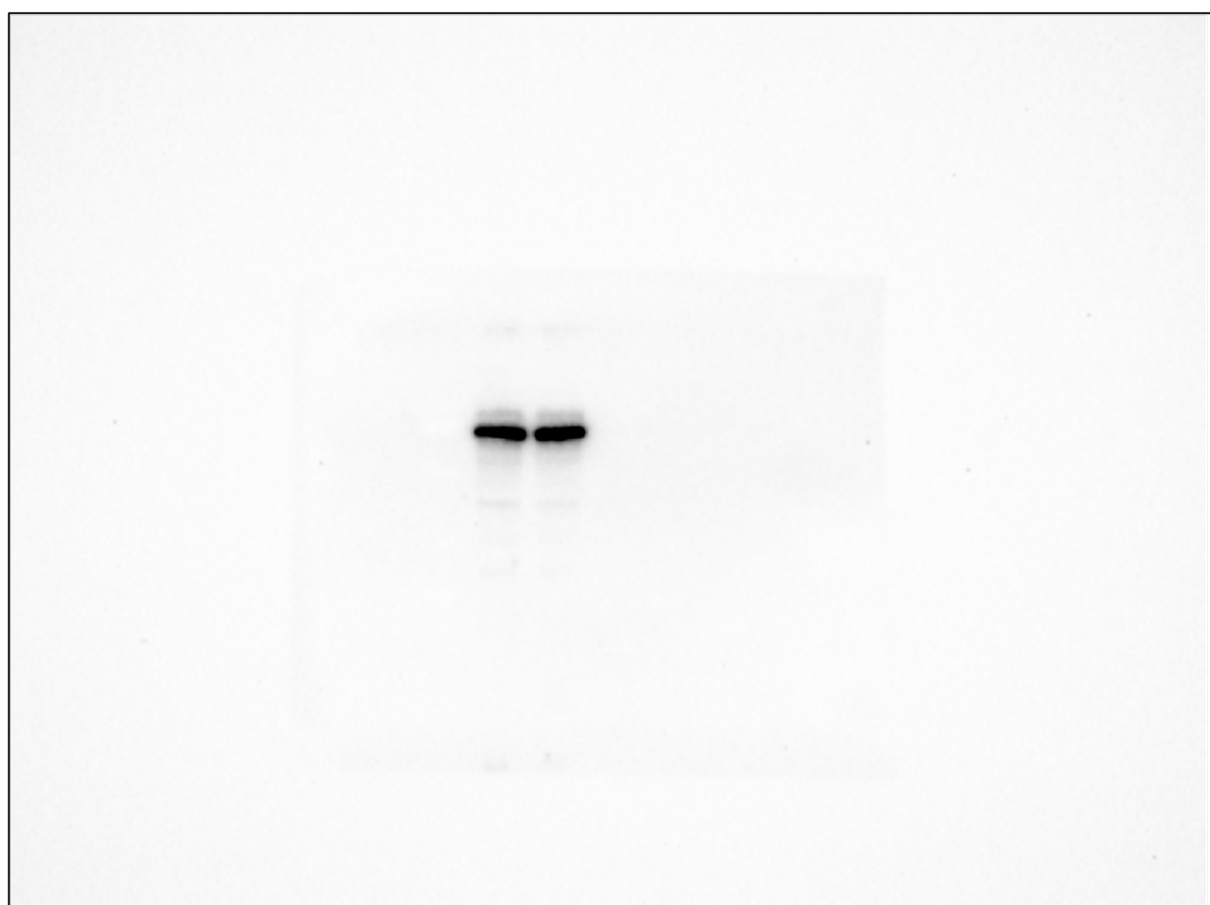

Supplement: Figure 1—figure supplement 3—source data 2. [file elife-66264-fig1-figsupp3-data2.pdf]

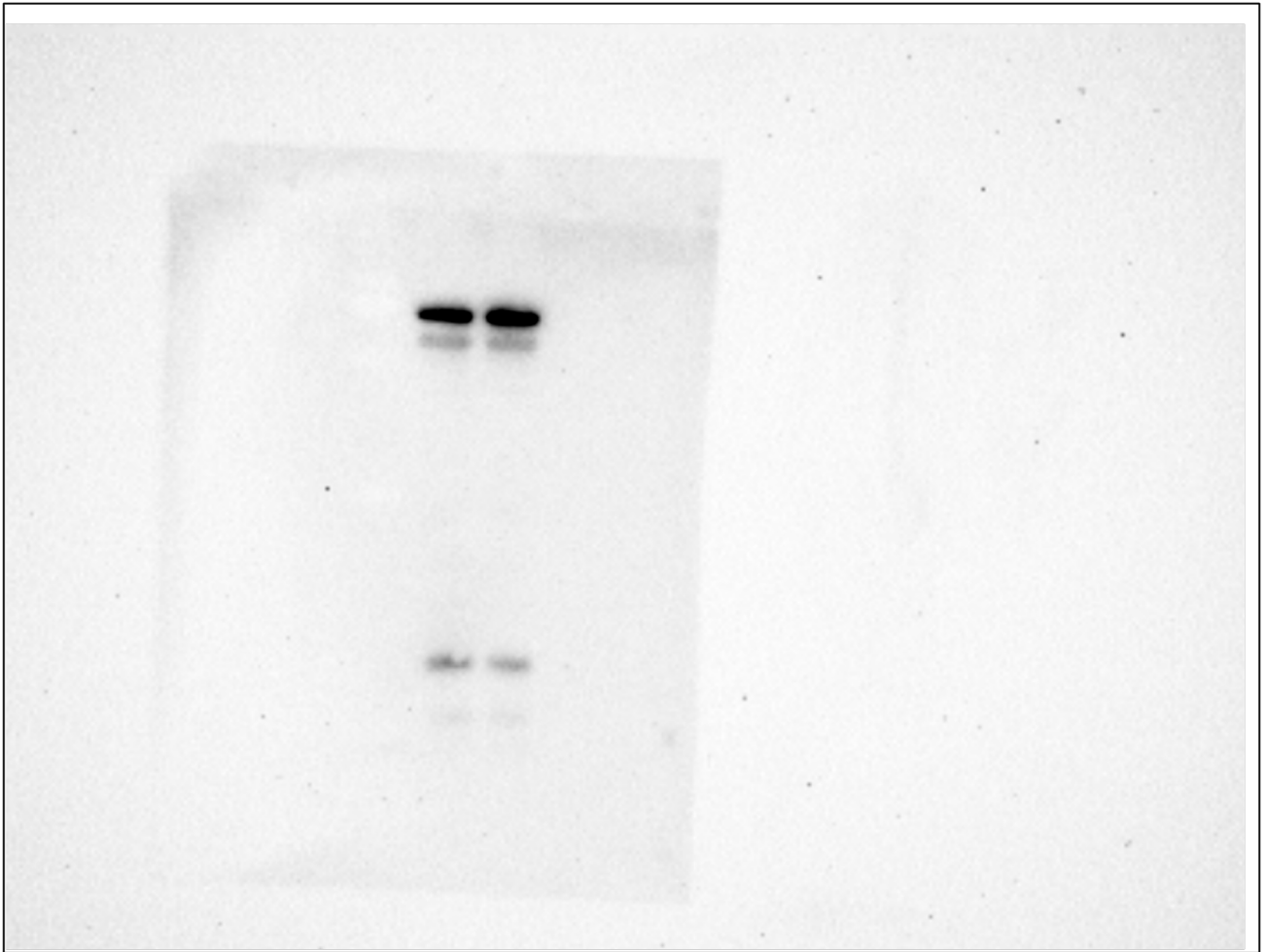

Supplement: Figure 1—figure supplement 3—source data 3. [file elife-66264-fig1-figsupp3-data3.pdf]

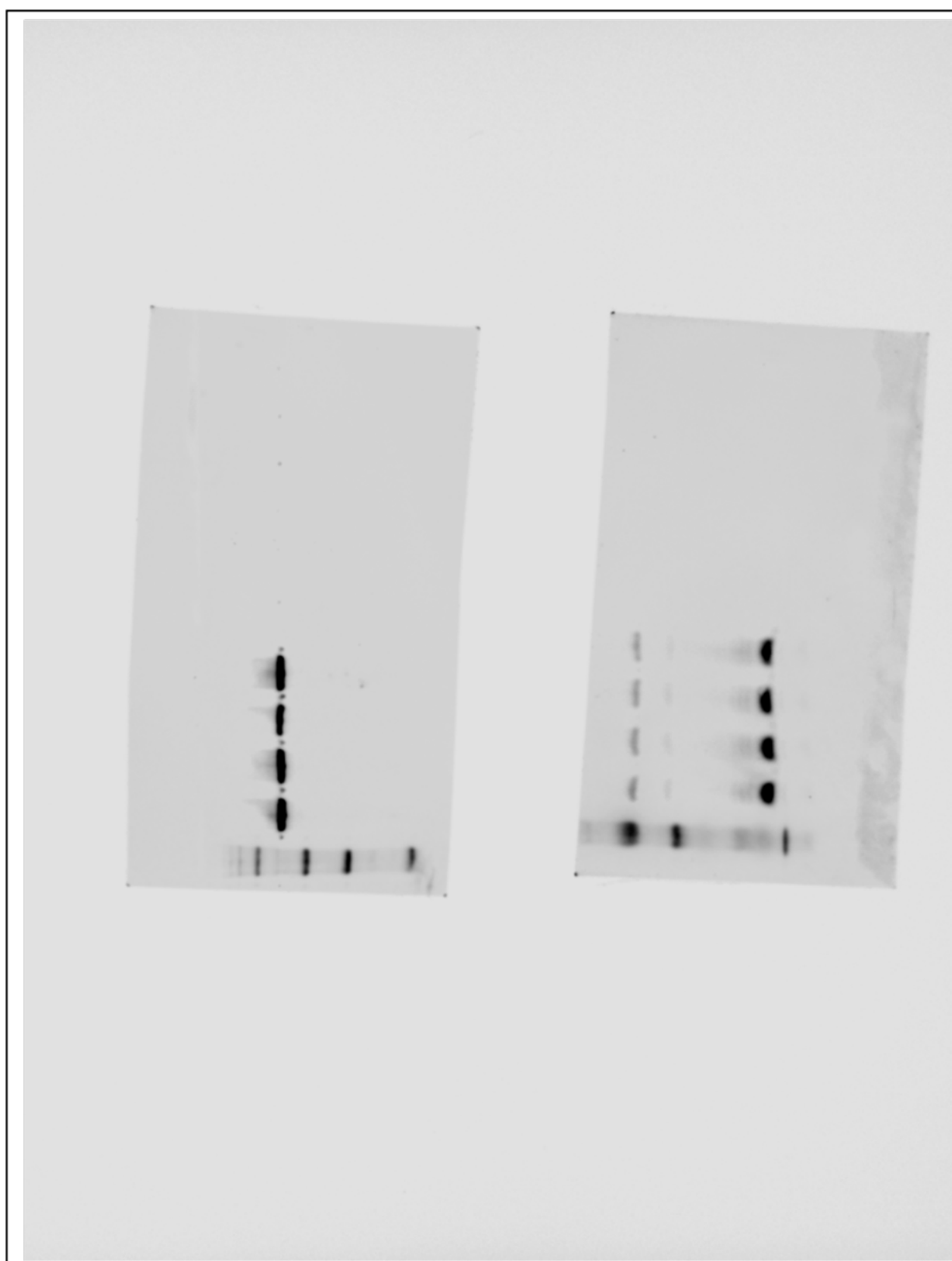

Supplement: Figure 1—figure supplement 5—source data 1. [file elife-66264-fig1-figsupp5-data1.pdf]

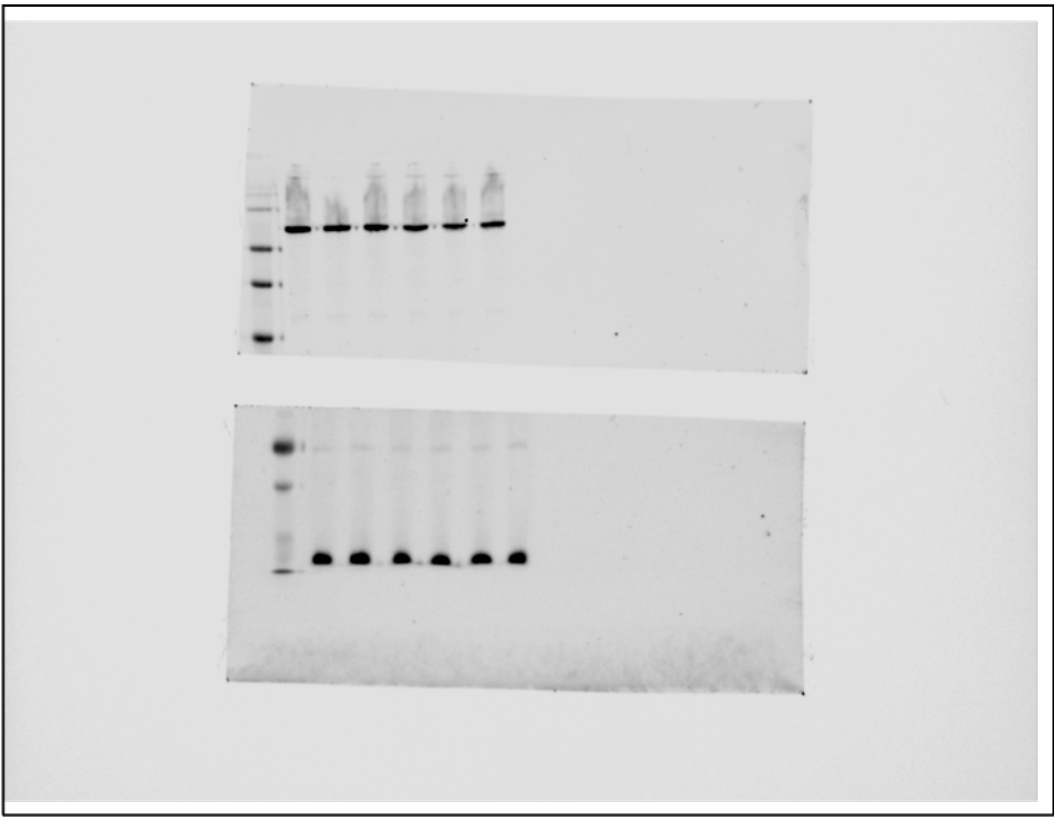

Supplement: Figure 1—figure supplement 5—source data 2. [file elife-66264-fig1-figsupp5-data2.pdf]

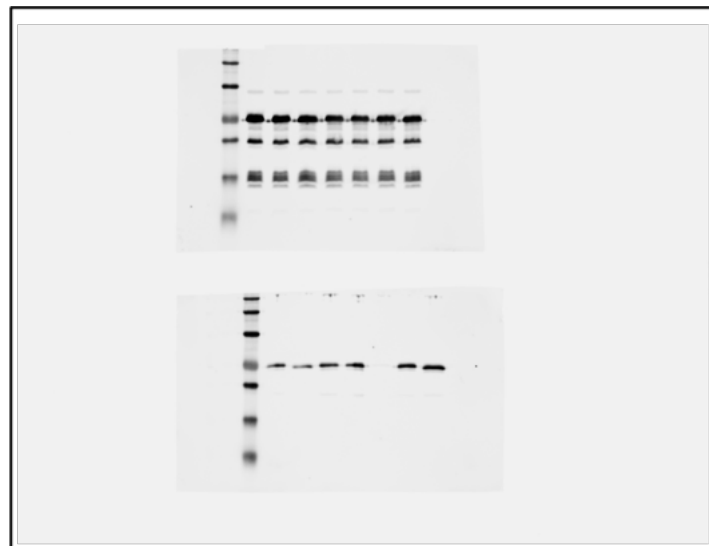

Supplement: Figure 2—source data 1. [file elife-66264-fig2-data1.pdf]

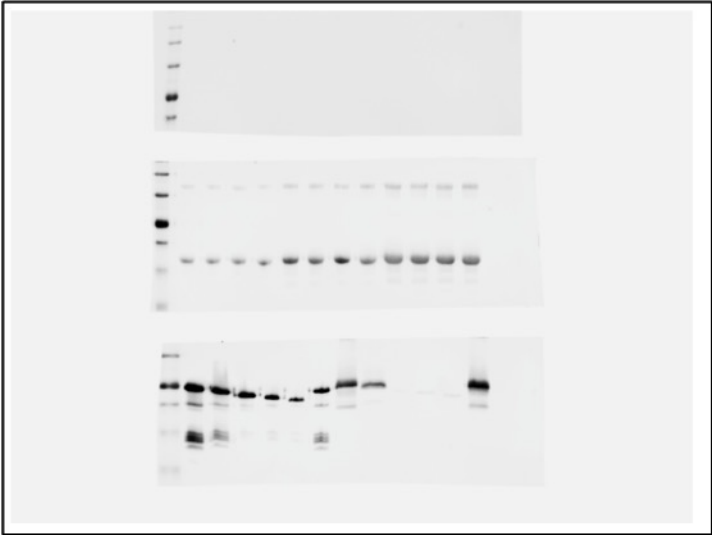

Supplement: Figure 2—source data 2. [file elife-66264-fig2-data2.pdf]

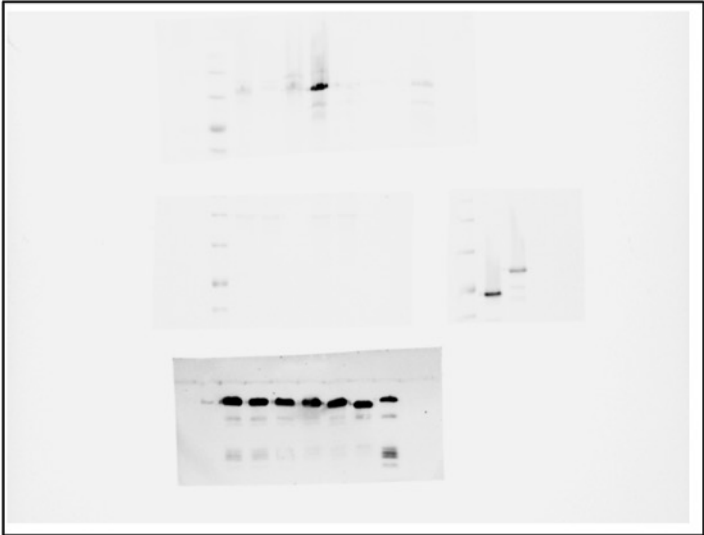

Supplement: Figure 2—source data 3. [file elife-66264-fig2-data3.pdf]

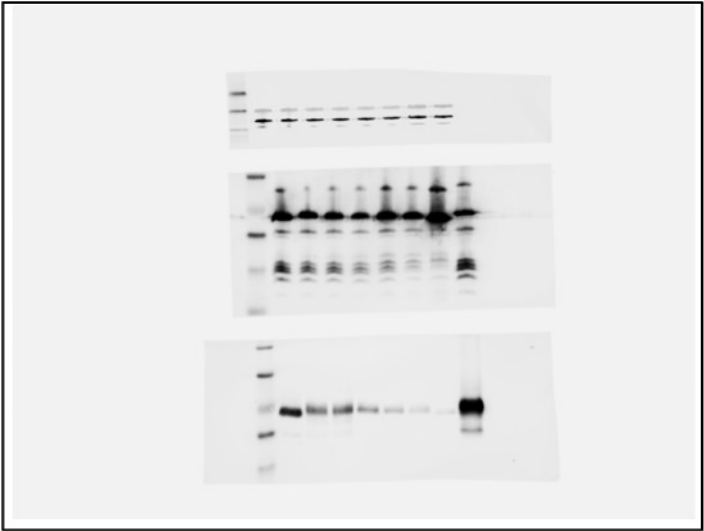

Supplement: Figure 2—source data 4. [file elife-66264-fig2-data4.pdf]

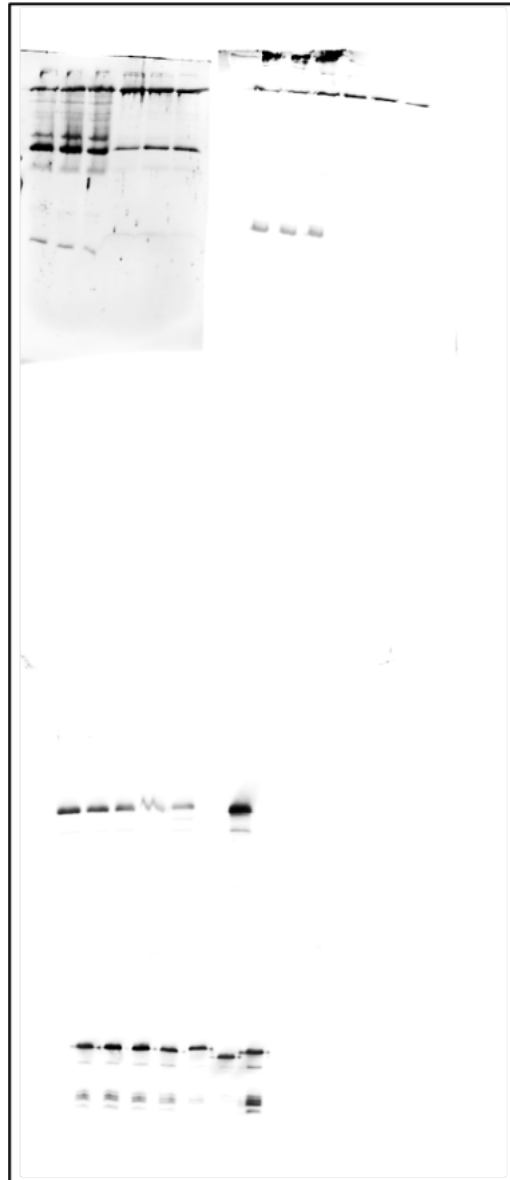

Supplement: Figure 2—source data 5. [file elife-66264-fig2-data5.pdf]

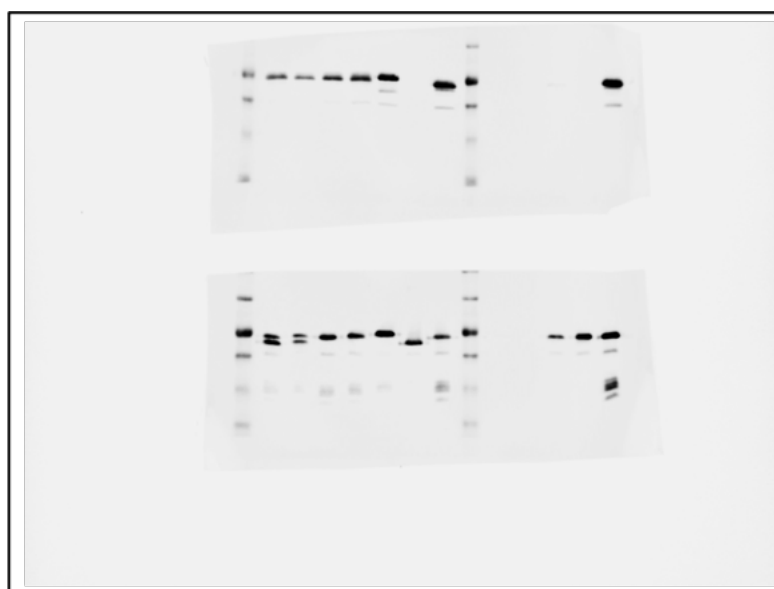

Supplement: Figure 2—source data 6. [file elife-66264-fig2-data6.pdf]

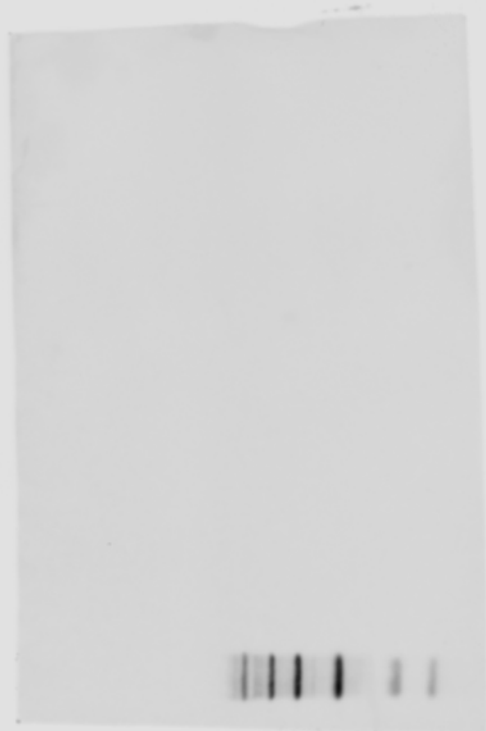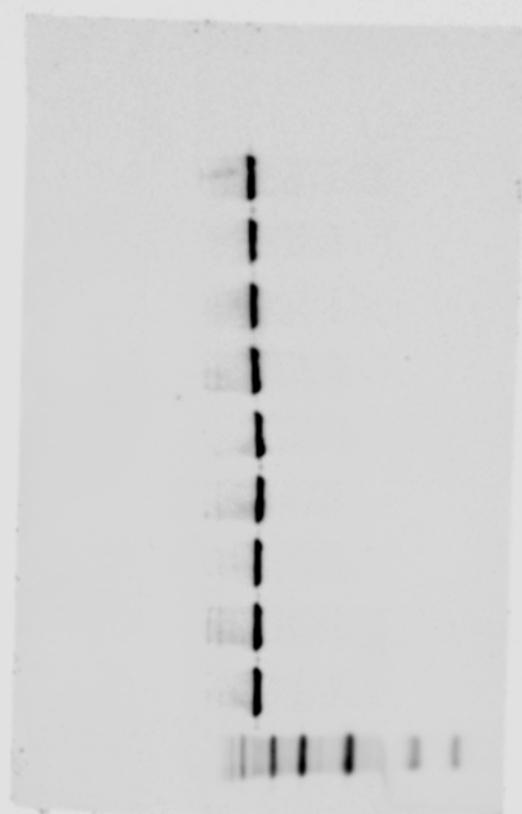

Supplement: Figure 2—figure supplement 2—source data 1. [file elife-66264-fig2-figsupp2-data1.pdf]

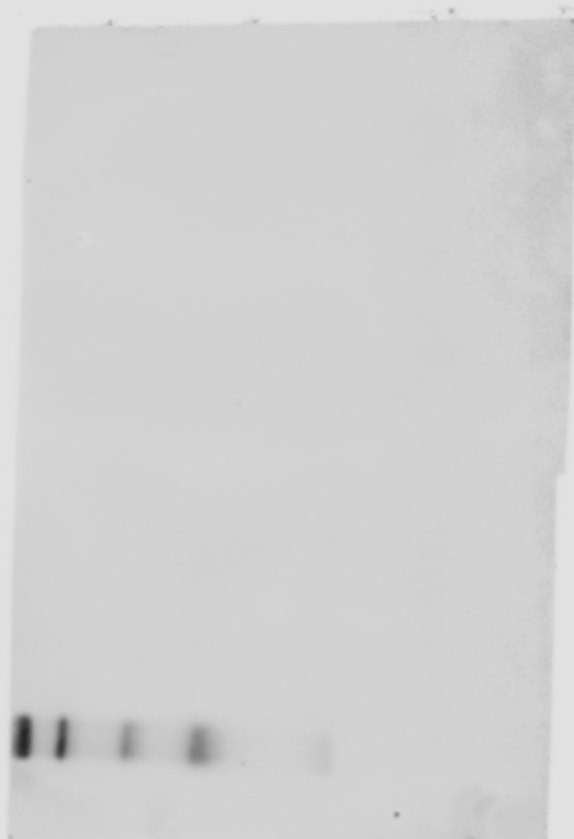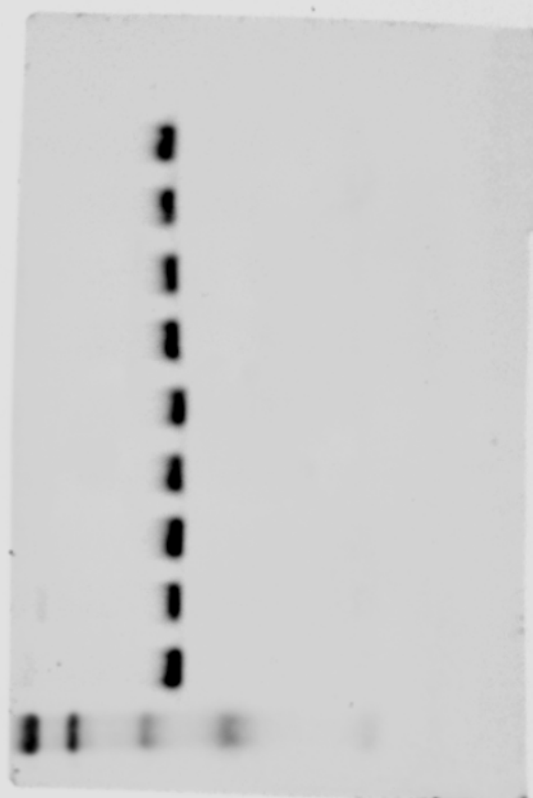

Supplement: Figure 2—figure supplement 2—source data 2. [file elife-66264-fig2-figsupp2-data2.pdf]

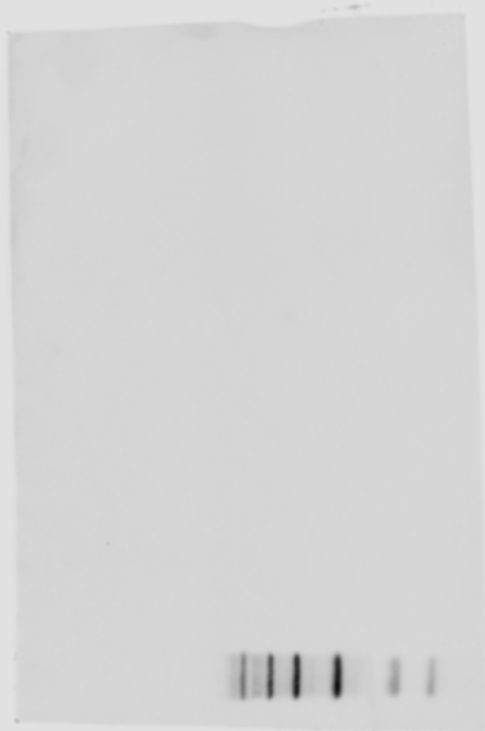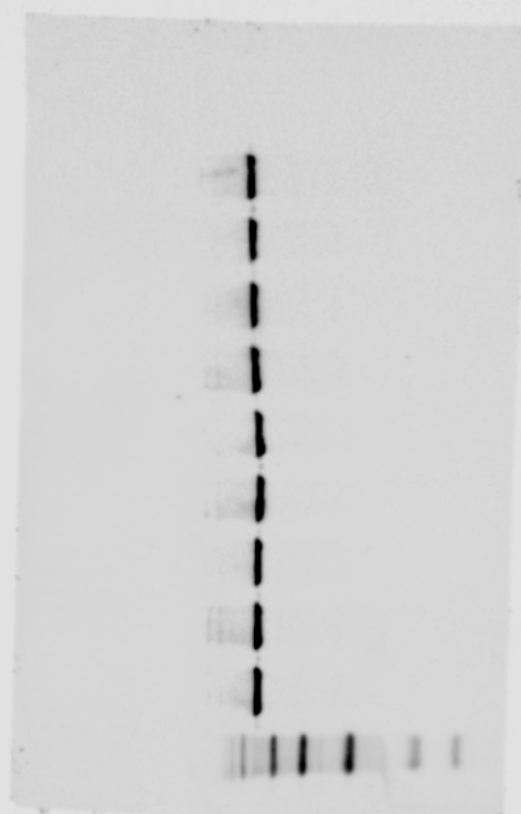

Supplement: Figure 2—figure supplement 2—source data 3. [file elife-66264-fig2-figsupp2-data3.pdf]

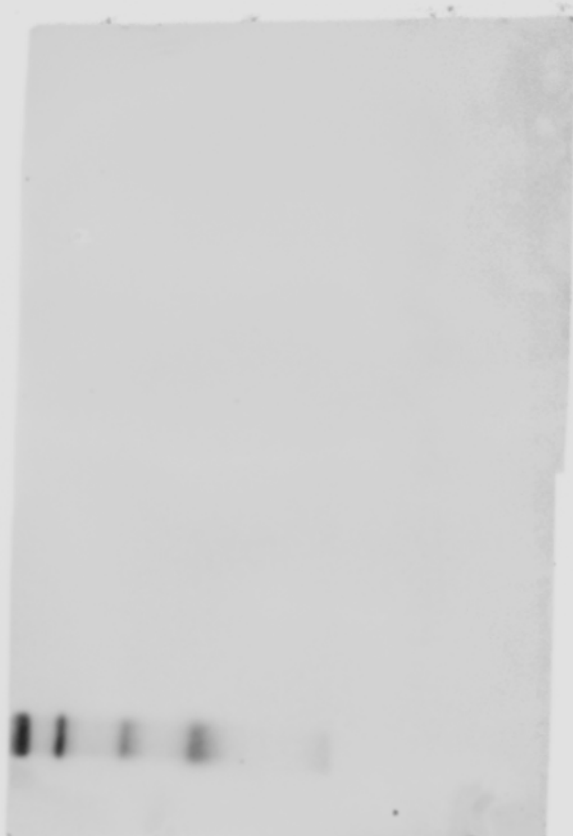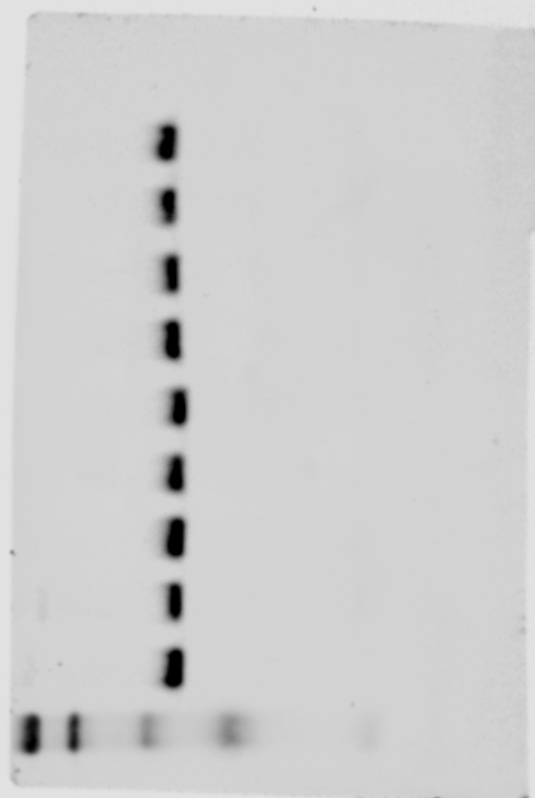

Supplement: Figure 2—figure supplement 2—source data 4. [file elife-66264-fig2-figsupp2-data4.pdf]

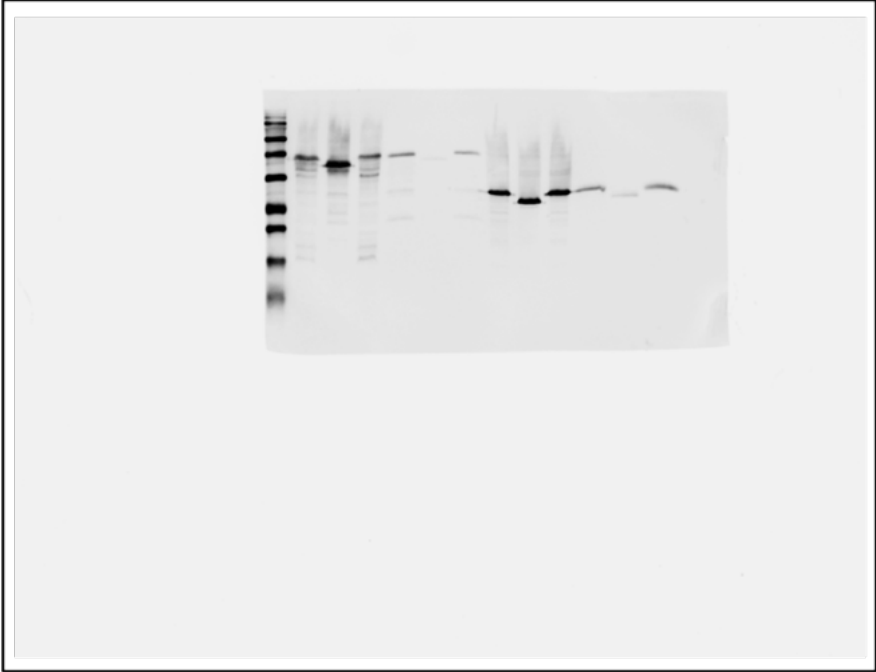

Supplement: Figure 3—source data 1. [file elife-66264-fig3-data1.pdf]

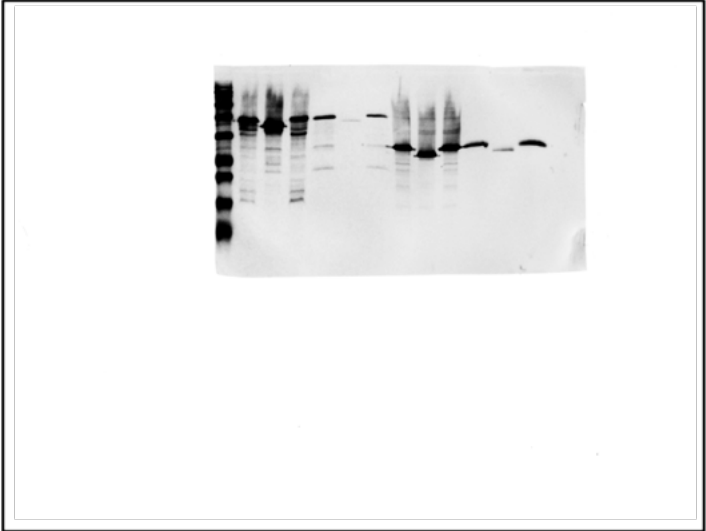

Supplement: Figure 3—source data 2. [file elife-66264-fig3-data2.pdf]

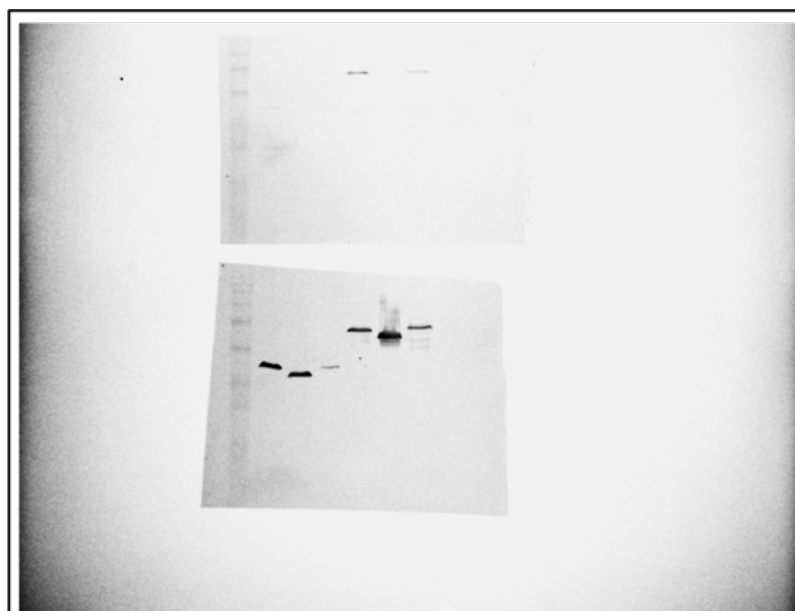

Supplement: Figure 3—source data 3. [file elife-66264-fig3-data3.pdf]

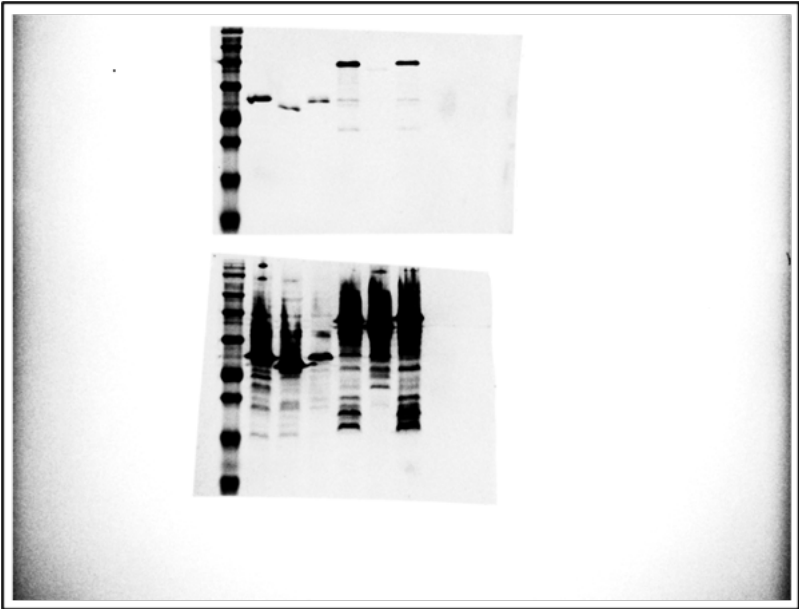

Supplement: Figure 3—source data 4. [file elife-66264-fig3-data4.pdf]

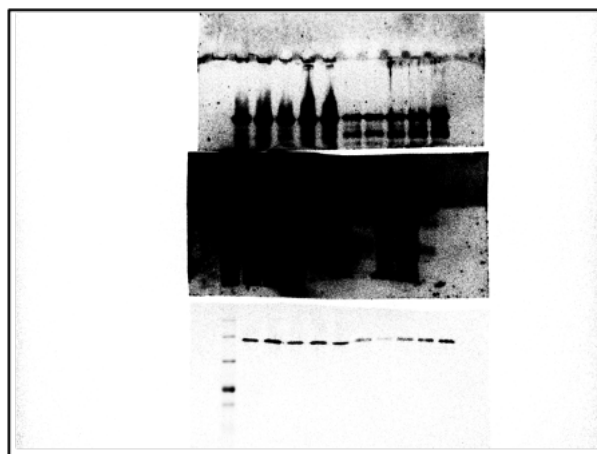

Supplement: Figure 4—source data 1. [file elife-66264-fig4-data1.pdf]

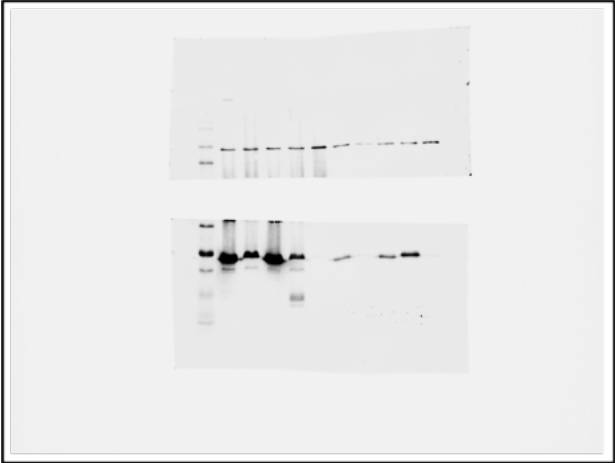

Supplement: Figure 4—source data 2. [file elife-66264-fig4-data2.pdf]

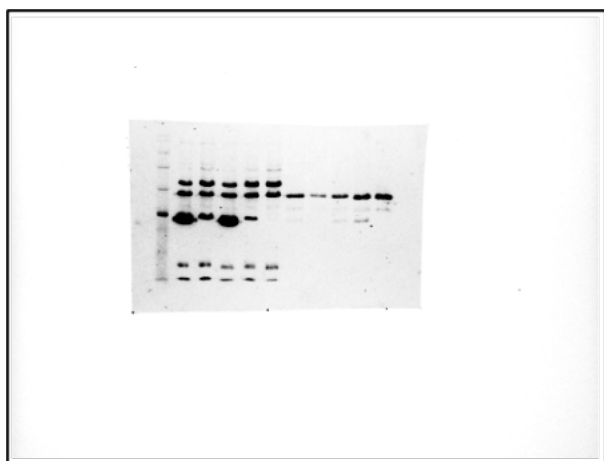

Supplement: Figure 4—source data 3. [file elife-66264-fig4-data3.pdf]

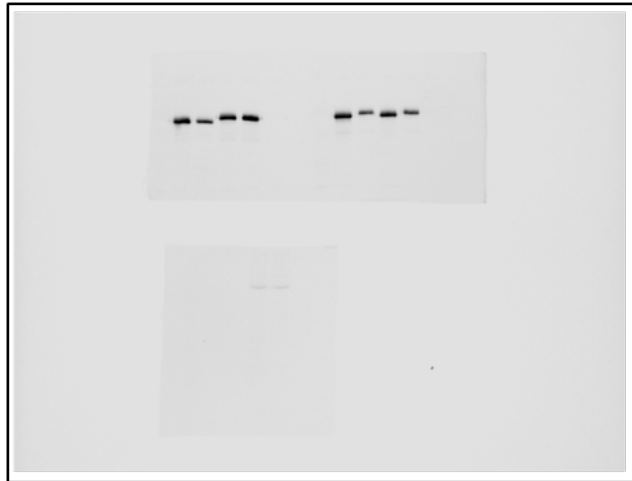

Supplement: Figure 4—source data 4. [file elife-66264-fig4-data4.pdf]

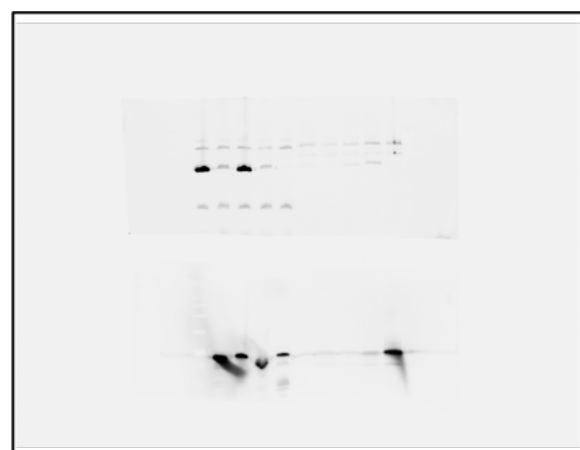

Supplement: Figure 4—source data 5. [file elife-66264-fig4-data5.pdf]

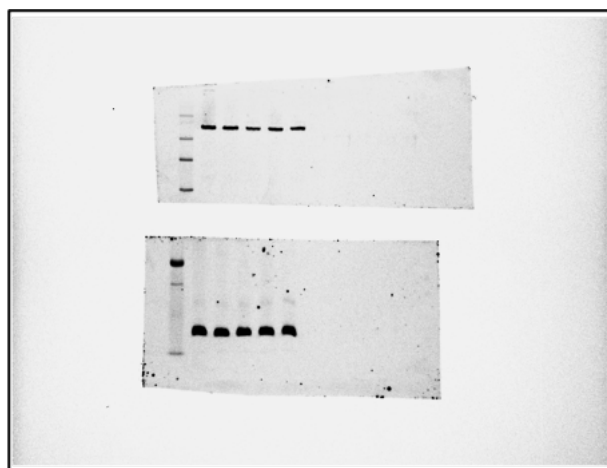

Supplement: Figure 4—source data 6. [file elife-66264-fig4-data6.pdf]

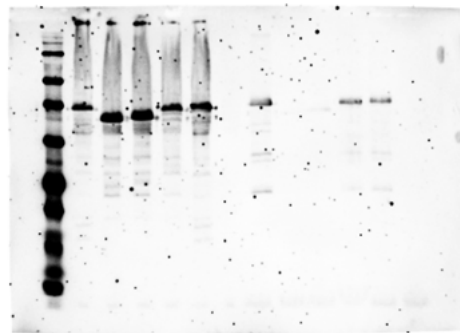

Supplement: Figure 5—source data 1. [file elife-66264-fig5-data1.pdf]

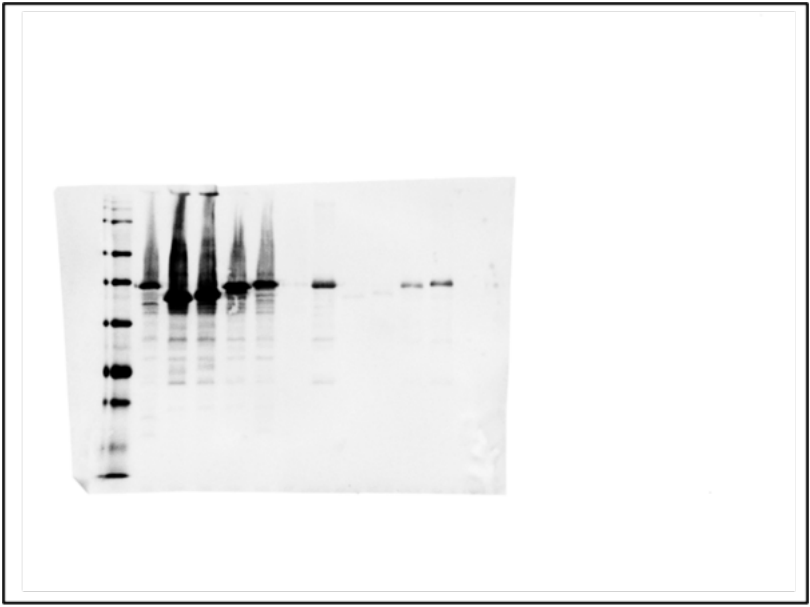

Supplement: Figure 5—source data 2. [file elife-66264-fig5-data2.pdf]

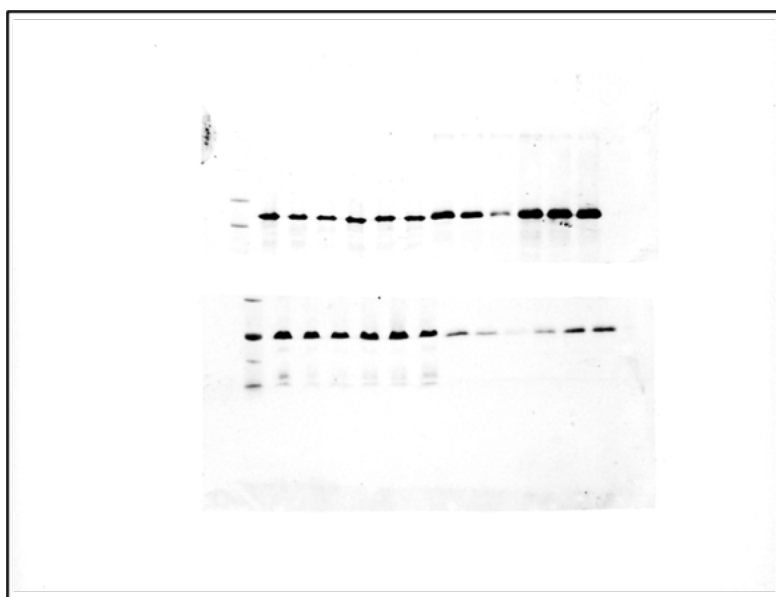

Supplement: Figure 5—source data 3. [file elife-66264-fig5-data3.pdf]

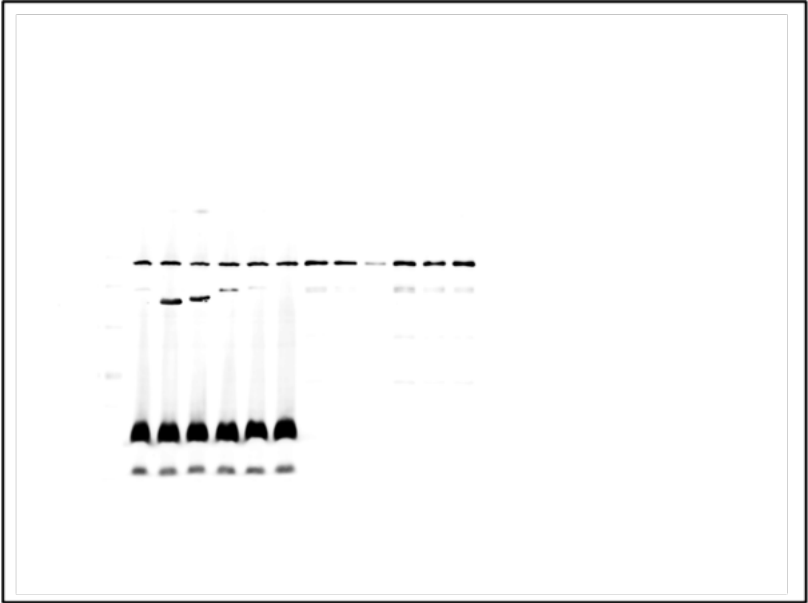

Supplement: Figure 5—source data 4. [file elife-66264-fig5-data4.pdf]

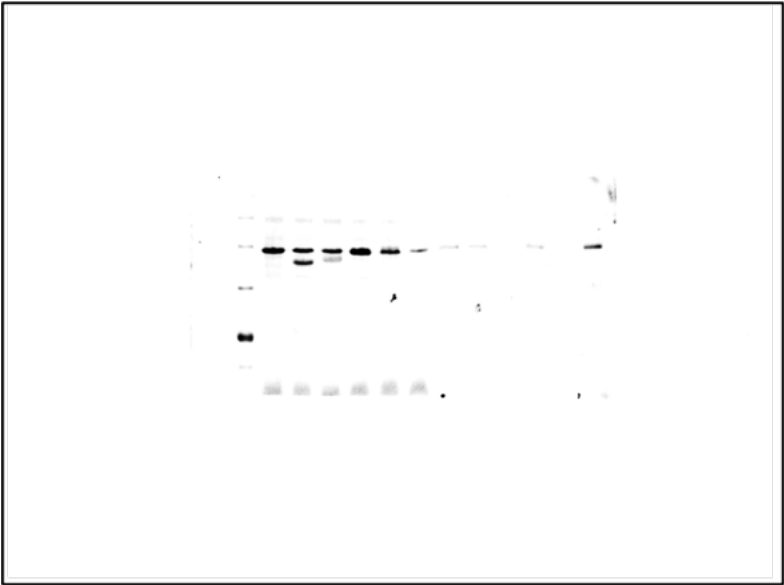

Supplement: Figure 5—source data 5. [file elife-66264-fig5-data5.pdf]

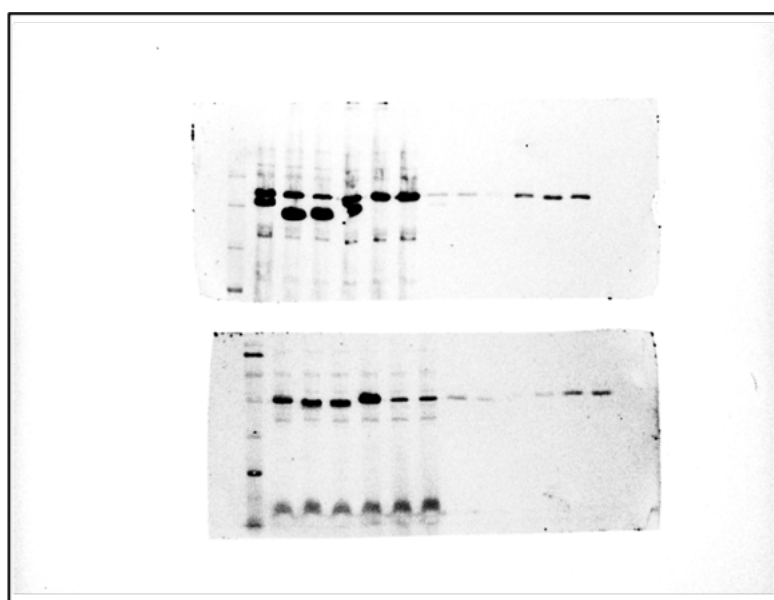

Supplement: Figure 5—source data 6. [file elife-66264-fig5-data6.pdf]

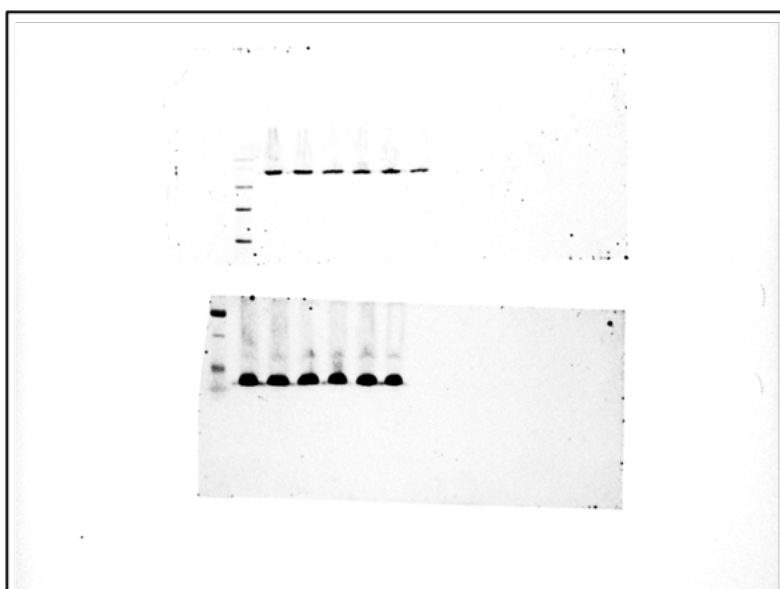

Supplement: Figure 5—source data 7. [file elife-66264-fig5-data7.pdf]

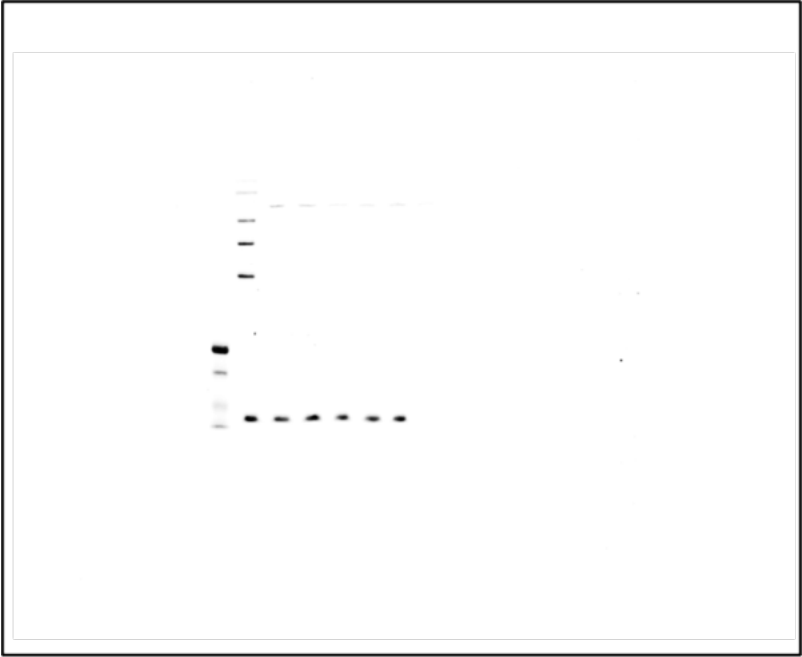

Supplement: Figure 5—source data 8. [file elife-66264-fig5-data8.pdf]

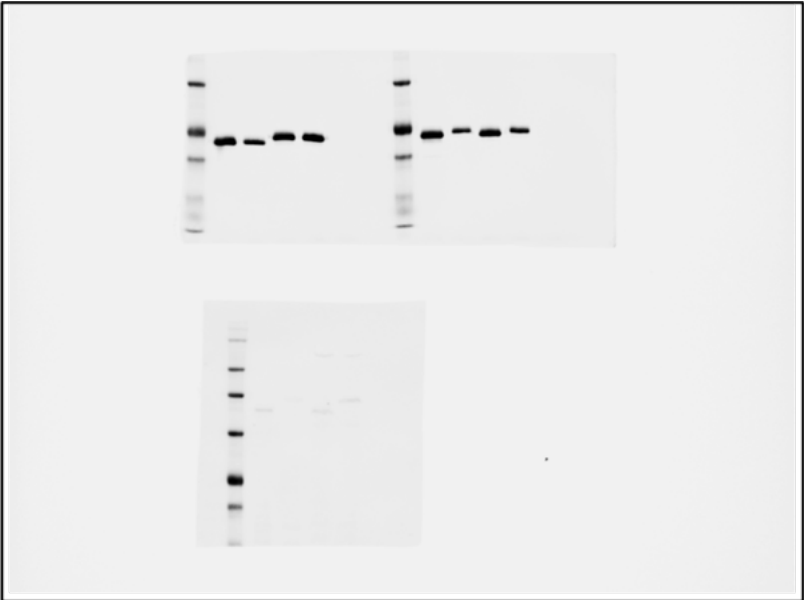

Supplement: Figure 5—source data 9. [file elife-66264-fig5-data9.pdf]

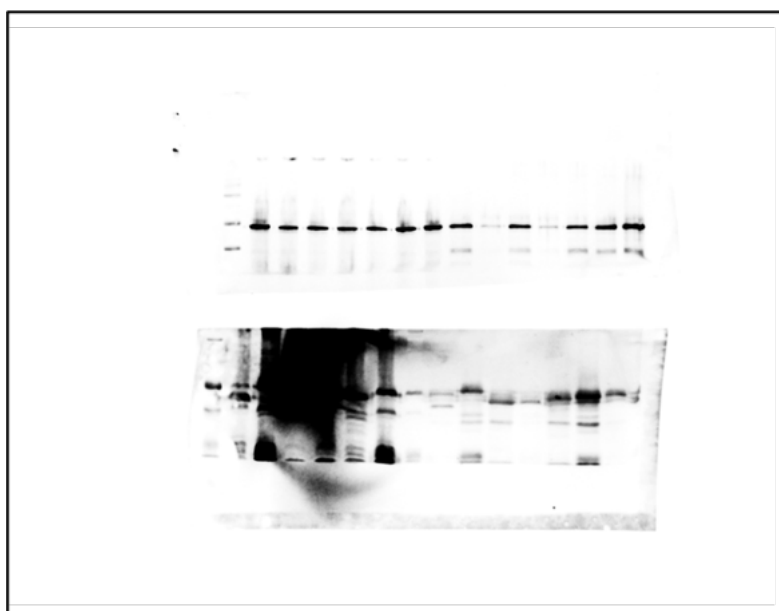

Supplement: Figure 5—source data 10. [file elife-66264-fig5-data10.pdf]

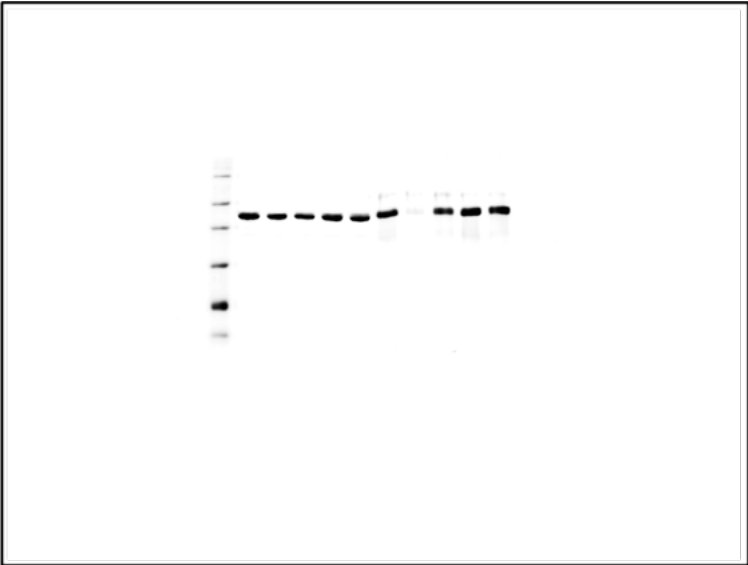

Supplement: Figure 5—source data 11. [file elife-66264-fig5-data11.pdf]

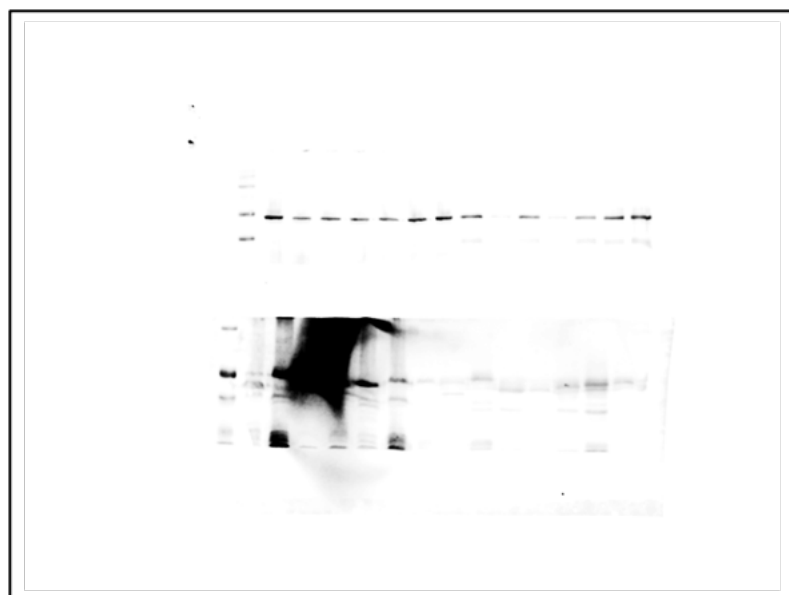

Supplement: Figure 5—source data 12. [file elife-66264-fig5-data12.pdf]

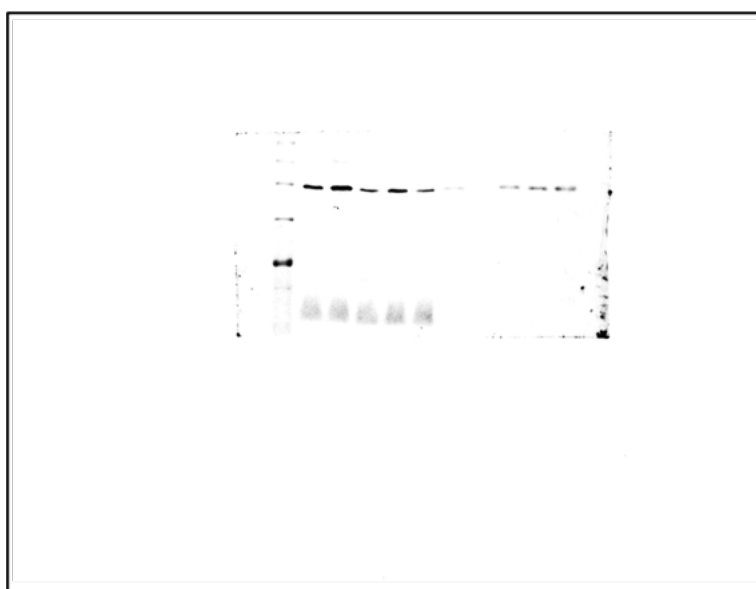

Supplement: Figure 5—source data 13. [file elife-66264-fig5-data13.pdf]

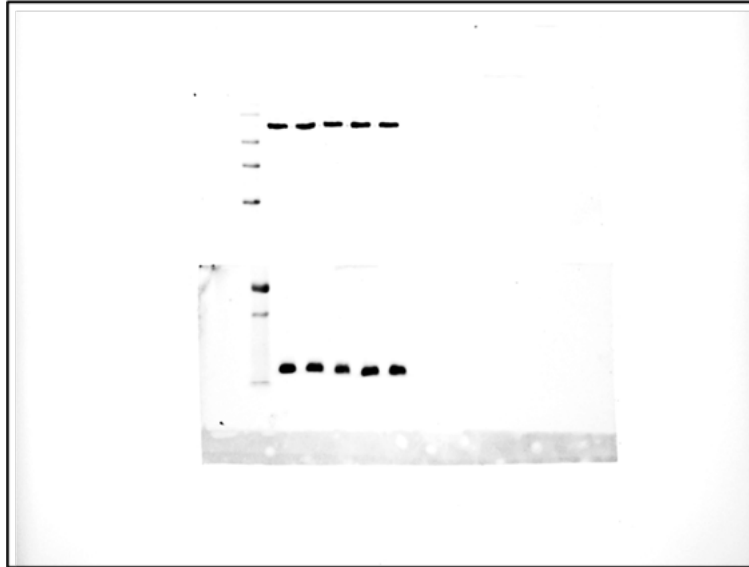

Supplement: Figure 5—source data 14. [file elife-66264-fig5-data14.pdf]

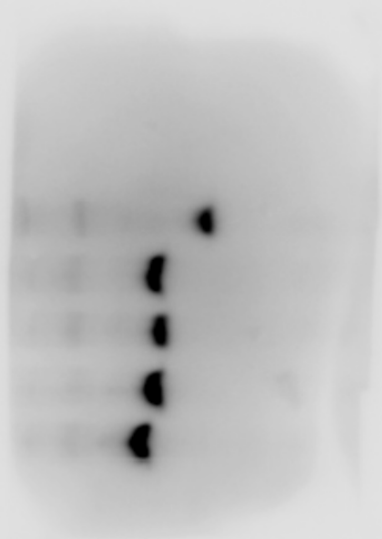

Supplement: Figure 6—figure supplement 1—source data 1. [file elife-66264-fig6-figsupp1-data1.pdf]

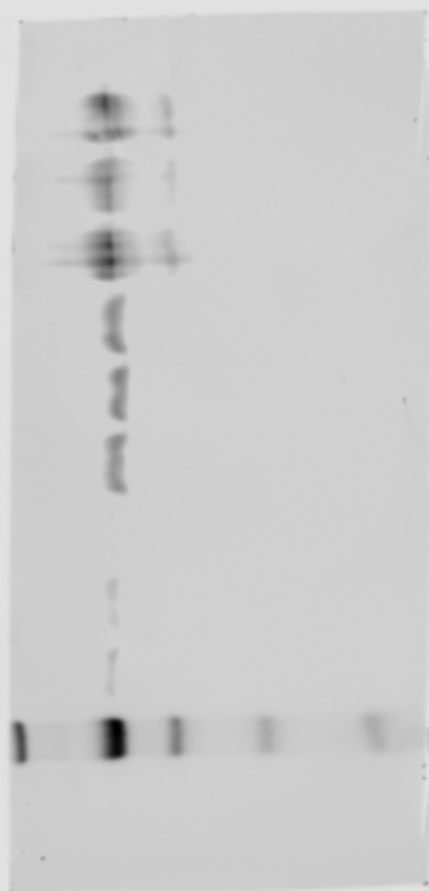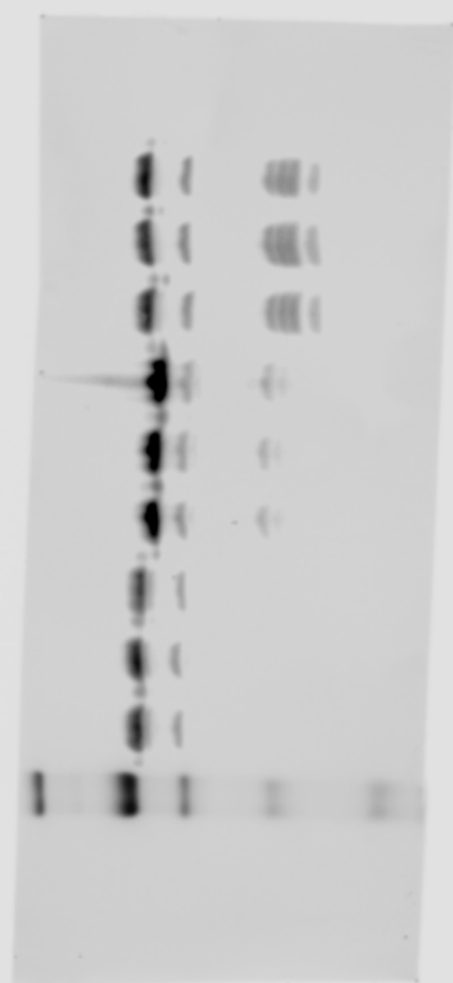

Supplement: Figure 6—figure supplement 1—source data 2. [file elife-66264-fig6-figsupp1-data2.pdf]
